# Rate of surgery in patients under treatment with a Chêneau light brace using the SRS inclusion criteria

**DOI:** 10.1186/1748-7161-7-S1-O45

**Published:** 2012-01-27

**Authors:** M Werkmann, HR Weiss

**Affiliations:** 1 Scoliocare Orthomed, Gensingen, Germany; 2 Orthopedic Rehabilitation Services, Gensingen, Germany

## Background

Studies investigating the outcome of conservative scoliosis treatment differ widely with respect to the inclusion criteria used. Prospective cohort studies are available using the SRS inclusion criteria for studies on bracing [1,2]. This seems to provide a great advantage to compare different strategies of bracing against each other. As we have gathered all data of the patients treated with a Chêneau light™ between June 2005 and November 2007 it was possible to identify the sample of patients fulfilling the SRS inclusion criteria from the whole sample.

## Materials and methods

34 patients (of 152) fulfilled the SRS inclusion criteria with an average age of 12.06 years (10 – 13 years), average Cobb angle of 31 degrees (25 – 40°), an average Risser stage of 0,35, average in-brace Cobb angele of 13° (= 59% of in-brace correction). There were 17 thoracic, 10 double major, 6 lumbar and 2 thoracolumbar curve patterns. After change of workplace of the second author the patients could not be followed up as planned. Therefore a telephone interview was performed by the first author.

## Results

28 patients (average age 16.5 years) have been reached, 9 of them were still under treatment. No patient has been operated (Rate of surgery 0%) and only one was not satisfied with cosmetic outcome of treatment.

## Discussion

Rate of surgery was far less than reported in recent studies using the same inclusion criteria even when all drop outs where rated as failures [1,2].

## Conclusions

Rate of surgery can be reduced with the help of Chêneau braces of the latest standard and satisfactory in-brace correction.
